# Letter to the editor regarding “Long-term mortality in pediatric sepsis: a systematic review and meta-analysis”

**DOI:** 10.1080/07853890.2026.2649387

**Published:** 2026-04-19

**Authors:** Kexin Ji, Xiaozheng Duan

**Affiliations:** a Changchun University of Chinese Medicine, No. 1035 Boshuo Road, Jingyue National High-Tech Industrial Development Zone,, Changchun, Jilin Province, China; b Affiliated Hospital of Changchun University of Chinese Medicine, No. 1478 Gongnong Road, Chaoyang District, Changchun, Jilin Province, China

Dear Editor,

We recently read with great interest the study by Lv et al. titled ‘Long-term mortality in pediatric sepsis: a systematic review and meta-analysis’ published in Annals of Medicine [1]. This pioneering work quantifies the long-term mortality burden in pediatric sepsis survivors, filling a critical knowledge gap in the field where research has long focused on in-hospital and short-term outcomes rather than post-discharge mortality risks. However, we would like to share some thoughts and suggestions for further discussion to enhance the depth and generalizability of the findings.

The rigorous methodological design, adhering to PRISMA guidelines and PROSPERO registration with a comprehensive global literature search, strengthens the validity of the pooled estimate. We particularly appreciate the first-ever meta-analytic finding of an 11% pooled long-term mortality rate (95% CI: 7–16%) in 11,318 pediatric sepsis patients, and the subgroup analyses by follow-up duration that confirm persistent mortality risks across different timeframes (14% for ≤3 months, 10% for 6–12 months). This advances clinical practice by highlighting the unrecognized vulnerability of pediatric sepsis survivors post-discharge and providing an evidence base for optimizing follow-up care protocols [2]. However, we note a few points worthy of discussion. First, the substantial heterogeneity (I^2^ = 98.2%) across included studies, stemming from regional healthcare disparities, varied study populations and follow-up protocols, may limit the direct generalizability of the pooled estimate; further subgroup stratification by economic development level and sepsis severity could clarify modifiable contributing factors. Second, the exclusion of non-English language studies and the small number of included studies (*n* = 6) may introduce potential selection bias, despite the limited publication bias identified *via* Egger’s test. Third, the absence of age-specific mortality trajectory analyses in the broad pediatric cohort (under 18 years) overlooks distinct physiological and clinical characteristics across age subgroups, which may hinder the identification of high-risk pediatric populations for targeted interventions [3].

Despite these limitations, this study makes a significant contribution to pediatric sepsis research. The leave-one-out sensitivity analysis and robust statistical approaches using the Freeman-Tukey double arcsine transformation add practical value by ensuring the reliability of the pooled mortality estimate. This work paves the way for evidence-based post-discharge surveillance and personalized care for pediatric sepsis survivors, which is crucial given that post-discharge mortality accounts for over half of all pediatric sepsis-related fatalities globally [4]. We commend the authors for their rigorous approach and meaningful findings, which address a long-neglected aspect of pediatric sepsis care aligned with global Sustainable Development Goals for child health. Further research with large, multi-center longitudinal cohorts, age-stratified analyses and cross-regional validation is warranted to identify modifiable risk factors and translate these findings into standardized pediatric sepsis follow-up guidelines.

Sincerely,
